# Follower-Centered Perspective on Feedback: Effects of Feedback Seeking on Identification and Feedback Environment

**DOI:** 10.3389/fpsyg.2017.01492

**Published:** 2017-09-01

**Authors:** Zhenxing Gong, Miaomiao Li, Yaoyuan Qi, Na Zhang

**Affiliations:** ^1^School of Business, Liaocheng University Liaocheng, China; ^2^Donlinks School of Economics and Management, University of Science and Technology Beijing Beijing, China; ^3^School of Economics and Management, Tsinghua University Beijing, China; ^4^Beijing Information Science and Technology University Beijing, China

**Keywords:** feedback seeking, feedback environment, identification, follower

## Abstract

In the formation mechanism of the feedback environment, the existing research pays attention to external feedback sources and regards individuals as objects passively accepting feedback. Thus, the external source fails to realize the individuals’ need for feedback, and the feedback environment cannot provide them with useful information, leading to a feedback vacuum. The aim of this study is to examine the effect of feedback-seeking by different strategies on the supervisor-feedback environment through supervisor identification. The article consists of an empirical study with a sample of 264 employees in China; here, participants complete a series of questionnaires in three waves. After controlling for the effects of demography, the results indicate that supervisor identification partially mediates the relationship between feedback-seeking (including feedback monitoring and feedback inquiry) and the supervisor-feedback environment. Implications are also discussed.

## Introduction

Toestablish and maintain their competitive advantage, enterprises must excel in various aspects, including planning, organizing, leading, controlling, and innovating (Sartori et al., 2013). Emphasizing the role of leaders in these aspects has become a trend in academic research and social practice (Avolio et al., 2009). By contrast, the role of employees is generally underestimated and ignored (Bjugstad et al., 2006). Due to the pressures posed by technological innovations and market competition, managers today are unable to handle business simply by relying on their entrepreneurship and past experience in the past; today, these leaders must rely on the enthusiasm and initiative of employees with preferable capacity, technology, and innovative ability (Bjugstad et al., 2006). Research on leadership has changed from the supervisor-centered viewpoint to the follower-centered viewpoint (Landino et al., 2006). Employees no longer accept executive instructions passively; in fact, they can impact their supervisors’ behavior with their initiatives (Bjugstad et al., 2006).

Ashford and Cummings (1983) proposed that employees can seek feedback via either the tactic of inquiry, which involves direct verbal requests for performance evaluations, or the more covert tactic of monitoring, which involves examining their environment for indirect feedback cues. As an active behavior, feedback-seeking is displayed by individuals actively seeking for valuable information to adapt to the development demands of the organization and individuals (Ashford et al., 2003); such feedback plays an active role in improving the employee’s clarity and performance (Anseel et al., 2015). In the feedback literature, researchers are encouraged to consider not only single feedback interventions but also the broader psychological feedback context in which these feedback interventions take place to fully understand how feedback affects work-related outcomes (Ashford et al., 2003; Steelman et al., 2004). Researchers have put forward the concept of the feedback environment, which refers to the contextual processes between a supervisor and a subordinate or two coworkers in the daily work environment (Steelman et al., 2004). Prior research has discussed the influence of the supervisor-feedback environment on employee feedback-seeking from a supervisor-centered viewpoint (De Stobbeleir et al., 2011) but ignores the active seeking of feedback by employees. Feedback-seeking can reduce the degree of information asymmetry in an organization and, in turn, influence the supervisor-feedback environment (Ashford et al., 2003). However, in the formation mechanism of the influencing supervisor-feedback environment, prior research pays attention to external feedback sources and factors of individuals that cannot be changed over a short period of time, such as knowledge, abilities, and values systems (Andrews and Kacmar, 2001), and regards individuals as objects passively accepting feedback. Discussions on feedback-seeking as a positive and active behavior influencing the path of feedback environment from the perspective of the feedback circuit are limited (Anseel et al., 2015). On the one hand, this limitation could contribute to external sources failing to realize the individuals’ need for feedback; on the other hand, it could also make individuals become aware that the feedback environment cannot provide them with useful feedback, leading to a feedback vacuum (De Stobbeleir et al., 2011). Therefore, discussing the role of feedback-seeking in the formation of the feedback environment is necessary.

In the current study, individual feedback-seeking cannot only stimulate a series of self-adjustments by providing the related information but also impact the viewpoint and behavior of the feedback provider. According to the viewpoint of inversed causes and effects described in the research on leadership by Shamir (2007), as followers of leaders, the characteristics and behaviors of employees can influence the characteristics and behaviors of supervisors. This finding reveals that managers tend to change their behaviors according to the behaviors of their subordinates and that feedback-seeking by employees is beneficial to improve the feedback environment. In this case, identification plays an important role as the mediator. According to previous research findings, the development of identification is composed of either top–bottom or bottom–top sense-collecting, sense-breaking, and sense-making, and the key element for identity development is the gathering of the related feedback information (Young and Steelman, 2014). More feedback-seeking behaviors lead to preferable development of feedback significance, which promotes consistency between behaviors and the external conditions. As the return on employee identity, more feedback-seeking behaviors lead to better quality and more-available feedback information from the external feedback source and a better feedback environment (Young and Steelman, 2014). In the feedback environment, feedback can be divided into two key feedback sources: coworkers and supervisors. Between coworkers and supervisors, supervisors play a larger role in influencing employee behavior (George and Zhou, 2007). Here, we prioritize the concept of the supervisor-feedback environment, similar to previous studies (Norris-Watts and Levy, 2004; Anseel et al., 2007).

Therefore, on the follower-centered perspective, this study tests the effect of feedback seeking by employee on supervisor feedback environment through supervisor identification.

## Theory and Hypotheses

### Feedback Seeking and Supervisor Feedback Environment

Ashford and Cummings (1983) define feedback-seeking as the conscious devotion of effort toward determining the correctness and adequacy of behaviors to attain valued end-states. The employee who actively seeks feedback more often has higher task clarity, is more skilled in tasks, and generally shows higher performance (De Stobbeleir et al., 2011). Ashford and Cummings (1983) thus proposed that employees can seek feedback using either the tactic of inquiry (feedback inquiry) or the more covert tactic of monitoring (feedback monitoring). In the former, individuals seek input into their performance by directly asking others for feedback; in the latter, individuals observe their own task progress and the actions of those around them to gain insights into various aspects of their performance (Ashford et al., 2003). Prior studies fail to distinguish feedback-seeking tactics or emphasize feedback inquiry (Linderbaum and Levy, 2010), and ignore discussions on distinguishing the roles of different feedback-seeking tactics and the related internal mechanism; thus, conflicting research results are common (Anseel et al., 2015). Morrison (2002) pointed out that the distinguishing measurement on feedback-seeking on the perspective of feedback sources and feedback-seeking tactics can solve this conflict. In China, a country in which a large power distance and high face culture are adopted, the observation type feedback-seeking method is more frequently adopted. Therefore, it is necessary to conduct distinguishing measurement on feedback-seeking of different tactics, which is more accurate.

On the basis of ontology, social psychology, and constructivism, Meindl (1995) proposed the follower-centered viewpoint. As followers of supervisors, employees manifest characteristics and behaviors of that can be considered independent variables; by comparison, the characteristics and behaviors of supervisors may be considered dependent or regulated variables. The follower-centered perspective focuses on the methods through which the characteristics, styles, role leading, self-identity, and behaviors of employees build the attitudes of supervisors, which means managers may comply with their subordinates and change their behaviors according to the behaviors of the latter (Plowman et al., 2007).

The feedback system in an organization is a dynamic process, and the feedback system structure is only the external expression of this dynamic process. The interaction between the elements of the feedback system is the internal basis for the existence of the system, which also constitutes the fundamental power of its evolution (Padilla et al., 2007). Creation of the environment creating and improvements in abilities form a dynamic process. Feedback-seeking can be built by the environment because the feedback environment can provide accurate and useful information and support for feedback-seeking; it can also encourage managers to improve the feedback environment. The feedback-seeking behaviors of employees are regarded as a representation of active work by supervisors and colleagues, who can then provide more supportive, accurate and honest feedback to encourage the feedback-seeking behaviors. Thus, the top–bottom feedback environment and bottom–top feedback-seeking mutually promote the effectiveness of the feedback system. At the theoretical aspect, researchers propose that uncertainty and role ambiguity cannot only influence feedback-seeking behaviors but also be influenced by these behaviors. However, empirical research on this topic is lacking (Anseel et al., 2015). Considering the above arguments, we offer the following assumption:

Hypothesis 1: Feedback monitoring would relate positively to supervisor feedback environment.
Hypothesis 2: Feedback inquiry would relate positively to supervisor feedback environment.

### Feedback Seeking and Identification

The existing research on significance construction focus on identification, and more observable expressions, such as feedback-seeking, appear during the interpersonal process (Press and Arnould, 2011). Feedback-seeking is the effort made by individuals to determine the acceptability of their performance (Anseel et al., 2015). Employees with more feedback-seeking behaviors intend to accept more accurate information about how to adapt to specific organizations or role relations, which will help them better understand their roles in the organization or their relations with others (Anseel et al., 2007). Given a better understanding of their role, employees can foster a closer relationship with their supervisors. Feedback-seeking is a strategy that allows employees to solve internal problems when they make clear about the identity (i.e., how much do I understand the target?). More feedback-seeking leads to more opportunities for other to point out how to adapt to the work situation and how to get along with other managers.

Organizations motivate managers to provide performance feedback to employees so that these employees can work better to achieve the organizational target. Emotions based on feedback are existent at the early stages of the manager–subordinate relation. Feedback seeking is correlated with the related identity, because it delivers dignity and respect, and admits the performance satisfaction, and emphasizes on the similarity between attitude and value, improving the identity of subordinates on the source of pride by supervisors. Feedback from manages is the most important information source that can be utilized by individuals to form their own viewpoint (Lord et al., 1999). The literature on socialization and role adoption explains how organizations determine organizational trust by utilizing institutionalized social strategies. By utilizing these strategies, organizations can clarify the identification of organizational members and employee experienced leaders to help to achieve the organizational target, to give out feedback (Pratt, 1998). Therefore, understanding why the feedback sought and received by individuals from senior organizational members (managers) can help the individual adapt to the role relation is easy. Individuals can seek and receive feedback from their managers and understand and benefit from the environment with target and expectation. Considering the above arguments, we offer the following assumptions:

Hypothesis 3: Feedback monitoring would relate positively to supervisor identification.
Hypothesis 4: Feedback inquiry would relate positively to supervisor identification.

### The Mediating Role of Identification

Employees can identify multiple sources in a working environment (Pratt, 1998). Individuals tend to assume low-order identities (e.g., supervisors and colleagues) and not high-order identities (e.g., organizations and society); here, the former has a better influence on identity, emotions, and behaviors than the latter (Ashforth et al., 2007). Identification occurs during the sense-collecting process and difference source. Sense-making, information integration, and difference elimination occur simultaneously. While seeking, receiving, explaining, and integrating feedback information from important sources of the environment, employees can identify these sources (Pratt, 1998). Qualitative research conducted by Vough (2012) identifies the types of information used during the identity process. For example, familiarity is correlated with identity. According to Vough (2012), employees who have knowledge and communicate with other members of the target team will identify the target faster. Individuals who are more knowledgeable on and master the daily activities of a certain type of work tend to identify a target through a familiar path. Thus, Vough (2012) postulates that people can identify other (please refer to similar logics) when feeling about the common grounds. If employees believe that a target employee has a similar ideology or world outlook, they tend to identify with this employee. Finally, according to Vough (2012), people also identify a target when they believe they can benefit from it (i.e., interest logic). If managers or colleagues provide another employee with constructive feedback, i.e., the feedback providing somebody with more social experience and tangible benefit, according to social exchange theory, he/she may understand the benefit of relations and depend on the organization more (Vough, 2012).

According to social identity theory, individuals tend to include themselves into corresponding groups to simply the world (Reid, 1987). Social identity theory implies that people possess a self-improvement mechanism (Anseel et al., 2007) and generally seek a positive identity or role in relation to the team to improve their individual position. Another motivation has been considered more important than self-identity and self-improvement, i.e., subordinate demand.” Subordinate demand describes people’s natural demand to become part of matters more powerful than them. Some scholars have conducted studies in this field, and new studies have verified the important role of subordinate demand in explaining why employees identify their working environment sources (Sluss and Ashforth, 2008). Several researchers believe that human beings possess a subordinate fundamental motivation that propels voluntary and non-voluntary behaviors. To meet demands, two conditions must be met: frequent and pleasant interaction must be present and the interaction should happen in stable and continuous-relation conditions (Baumeister and Leary, 1995). Feedback-seeking can result in self-adjustment with the sought information, and individuals can feel about the sense of work subordination after achieving the requirements of others.

Under the background of rapid changes in organizations and the requirement that employees achieve continuous adaptation, identification is clearly important. According to existing research, identification can increase performance (Van Knippenberg, 2000) and reduce separation rates (Mael and Ashforth, 1995). The relation between subordinates and supervisors influences the motivations and performance of the former, and the identification of the latter deepens this relation (Sluss and Ashforth, 2008). Because supervisor identification reflects the individual attachment of subordinates to supervisors, and the identity from supervisors of high level means higher individual attachment level of subordinates to supervisors. Subordinates with a relatively high level of supervisor identity will pay more attention and strive to maintain and develop the supervisor–member relation. Supervisor identification can motivate subordinates to realize the value system in their self-concept which is similar to supervisors, and they even long for changing self-concept to achieve more similarities between their value system and belief and supervisors. According to research findings, supervisor identification influences the experience and behavior of individuals, as well as the adaptation of newcomers (Sluss and Ashforth, 2008). As the return to supervisor identification, more feedback-seeking behaviors will lead to higher quality and more-accessible feedback information and a better feedback environment from external feedback sources (Young and Steelman, 2014) to improve management validity. Considering the above arguments, we offer the following assumption:

Hypothesis 5: Supervisor identification would mediate the relationship between feedback monitoring and supervisor feedback environment.
Hypothesis 6: Supervisor identification would mediate the relationship between feedback inquiry and supervisor feedback environment.

## Materials and Methods

### Participants and Procedures

Participants who provide valid responses for three waves of study consist of 325 full time employees from 13 industry firms in five provinces of China by contacted via HR-managers. Every participant does not need to fill their name but only write last eight number of their ID card. This number is treated as corresponding standard for every participant.

For clarifying the causal relationships among variables, this study uses longitudinal research design to collect data like prior longitudinal research on feedback environment (Gabriel et al., 2014), Employees provide data at three time points for 3 months apart to help mitigate concerns associated with having same-time, same-source data. Participants are instructed to complete the questionnaire at three times points. Survey 1 includes feedback seeking and demographic measures, and was launched at the end of January 2015. There are 307 valid responses. Survey 2 assesses supervisor identification, and was started at March 2015. There are 296 valid responses. Survey 3 measures supervisor feedback environment and was started at mid June 2015. At last there are 264 valid questionnaires (completed at all phases of the study and without invalid answers, such as only writing one score for the whole questionnaire, etc.) received.

Demographic information indicates that there are 145 male employees and 119 female employees in the sample. More than half of the employees are 20–30 years of age, 95% of the participants are under 40 years old. The average organizational tenure is 7.03 years. Sixty-three percent of the participants hold a bachelor’s degree or above.

### Ethics Statement

This study is reviewed and approved by American Psychological Association Ethics Committee Rules and Procedures, APA Ethics Committee with written informed consent from all participants. All participants have given written informed consent in accordance with the Declaration of Helsinki.

### Instruments

The measure items used in the present study are primarily developed in English; thus, to ensure cross-linguistic equivalence, we translate all scale items into Chinese and then translate them back into English by means of two bilingual (English–Chinese) professional translators (Brislin, 1980). In order to try not to negatively affect the validity and reliability of the original instruments translated into the new ones, we followed the literature on the matter (Sartori and Pasini, 2007) and do as follows.

#### Feedback Seeking

Ashford and Cummings (1983) propose that employees can seek feedback using either the tactic of inquiry (feedback inquiry), which involves direct verbal requests for performance evaluations, or the more covert tactic of monitoring (feedback monitoring), which involves examining their environment for indirect feedback cues. Two items are created to assess the frequency with which individuals seek feedback by inquiry about performance behaviors (Callister et al., 1999). Responses to these items are measured on a 5-point response scale, ranging from never to frequently. Confirmatory factor analysis (CFA) is conducted to examine whether the factor structure of this scale can be supported by current data. Fit indicators based on the data from Wave 1 (*N* = 264) are: χ^2^ = 72.63, *df* = 34, χ^2^/*df* = 2.14; comparative fit index (CFI) = 0.96, incremental fit index (IFI) = 0.97, root mean square error of approximation (RMSEA) = 0.06, SRMR = 0.05. The reliability of the frequency of feedback seeking scale is α = 0.91. Two items asked respondents to report how frequently they seek feedback by monitoring about performance behaviors (Callister et al., 1999). Responses to these items are measured on a 5-point response scale, ranging from never to frequently. Fit indicators based on the data from Wave 1 (*N* = 264) were: χ^2^ = 66.88, *df* = 30, χ^2^/*df* = 2.23; CFI = 0.99, IFI = 0.98, RMSEA = 0.05, SRMR = 0.02. The reliability of the frequency of feedback seeking scale was α = 0.89.

#### Supervisor Identification

Supervisor identification is measured using a modified version of Edwards and Peccei’s (2007) 6-item scale that is originally developed to measure organizational identification. This instrument is modified by changing the referents to supervisor to measure supervisor identification. In organizational identification research, changing the scale’s referent from organization to another specific target that the researcher wants to measure is common practice (Young and Steelman, 2014). Fit indicators based on the data from Wave 2 (*N* = 264) are: χ^2^ = 8.12, *df* = 5, χ^2^/*df* = 1.62; CFI = 0.99, IFI = 0.98, RMSEA = 0.05, SRMR = 0.03. The Cronbach’s α for the measure of supervisor feedback environment is 0.85.

#### Supervisor Feedback Environment

We measure the supervisor feedback environment using Steelman et al.’s (2004) scale. Given that we are primarily concerned with the supervisor feedback environment, only the 32 supervisor-focused items are used. All questions are on a 7-point scale (1 = “strongly disagree”; 7 = “strongly agree”) and averaged to create a composite score. This Likert scale assesses each feedback environment dimension, including Source Credibility, Feedback Quality, Feedback Delivery, Favorable Feedback, Unfavorable Feedback, Source Availability, and Promotes Feedback Seeking. Fit indicators based on the data from Wave 3 (*N* = 264) are: χ^2^ = 724.42, *df* = 168, χ^2^/*df* = 4.31; CFI = 0.93, IFI = 0.92, RMSEA = 0.07, SRMR = 0.03. The Cronbach’s α for the measure of supervisor feedback environment is 0.94.

#### Controls

We measure and control for the effects of participants’ age, job tenure, gender (1 = male, 2 = female), education level (1 = associate degree or below, 2 = Bachelor, 3 = Master or above).

### Data Analysis

To examine the convergent and discriminant validity of the key variables, we employ structural equation modeling to conduct the discrimination validity of CFA using AMOS 21.0.

Descriptive analyses are performed to describe the participants’ demographic characteristics. Pearson correlation analysis is used to determine the relationships among the variables. Simple multiple linear regression is used to determine the proportion of variance using SPSS 21.0.

To test mediation, we adopted the procedure proposed by Preacher and Hayes (2008). According to their suggestions, there are three criteria to justify a mediation effect. First, the independent variable should be significantly correlated with mediator variable. Second, after the effect of the independent variable toward dependent variable is controlled, the correlation between mediator variable and dependent variable should be significant. Finally, the indirect effect of independent variable on dependent variable must be significant. Before the analyses, all continuous predictors are well-centered. To calculate the indirect effects, this study utilizes the SPSS macro PROCESS (Hayes, 2013).

## Results

The main purpose of this study is to demonstrate that the relationship between the feedback seeking and supervisor feedback environment is mediated by supervisor feedback environment. This study assesses overall model fit by goodness-of-fit indices including the CFI, IFI, and RMSEA. A reasonable model fit is indicated when the CFI and IFI are above 0.90 and the RMSEA is below 0.08 (Hu and Bentler, 1998).

For testing the discrimination validity of the CFA, this study compares two three-factor models (Models 1 and 3) with two one-factor models (Models 2 and 4). The results show that the three-factor model fits the data better than other nested models (see **Table 1**), indicating that the three variables show good discriminating validity. In summary, the CFA results suggest that the respondents could clearly distinguish the constructs under study.

**Table 1:** Confirmatory factor analysis of discrimination validity.

Model	Factor loaded	χ^2^	*df*	χ^2^*/df*	CFI	IFI	RMSEA
Model 1	Three factors: FM, SI, and SFE	169.92	72	2.36	0.92	0.91	0.07
Model 2	One factor: FM, SI, and SFE are combined into one factor	233.25	75	3.11	0.81	0.77	0.09
Model 3	Three factors: FI, SI, and SFE	177.12	72	2.46	0.95	0.93	0.08
Model 4	One factor: FI, SI, and SFE are combined into one factor	240	75	3.20	0.80	0.76	0.10


*n = 264. FM, feedback monitoring; FI, feedback inquiry; SI, supervisor identification; SFE, supervisor feedback environment*.

**Table 2** presents the means, standard deviations, and correlations among the study variables. An inspection of the correlations reveals that feedback monitoring and feedback inquiry relate positively to supervisor feedback environment (*r* = 0.44, *p* < 0.01; *r* = 0.41, *p* < 0.01) and supervisor identification (*r* = 0.47, *p* < 0.01; *r* = 0.36, *p* < 0.01). The results also indicate that supervisor identification is positively correlated with supervisor feedback environment(*r* = 0.40, *p* < 0.01). Some demographic variables are related to feedback seeking, supervisor identification and supervisor feedback environment, and controlling demographic variables effect can make the results be more clarity.

**Table 2:** Means, standard deviations, and correlations of all measures.

	Mean	*SD*	1	2	3	4
1. Feedback monitoring	5.1	1.29	–			
2. Feedback inquiry	4.55	1.55	0.60^∗∗^	–		
3. Supervisor identification	5.12	1.12	0.47^∗∗^	036^∗∗^	–	
4. Supervisor feedback environment	4.76	0.63	0.44^∗∗^	0.41^∗∗^	0.40^∗^	–
5. Gender	–	–	0.22^∗^	-0.05	-0.14^∗^	0.07
6. Age	2.21	1.09	0.27^∗^	0.14^∗^	0.1	0.02
7. Job tenure	2.49	1.12	0.08	0.17^∗^	0.19^∗^	0.18^∗^
8. Education	–	–	0.14^∗^	0.03	0.08	-0.04


*n = 264; ^∗^p < 0.05, ^∗∗^p < 0.01*.

Further analyses are conducted to better estimate the overall contribution of feedback seeking (including feedback monitoring and feedback inquiry) in predicting supervisor feedback environment as well as the mediation role of supervisor identification. To examine whether supervisor identification as a mediator for the relations between feedback seeking and supervisor feedback environment, we adopt the procedure proposed by Preacher and Hayes (2008).

When the independent variable is feedback monitoring, as showed in **Table 3**, after controlling for the effect of participants’ demographics (gender, age, education, and tenure), feedback monitoring significantly predicts supervisor identification (Model 1: β = 0.35, *p* < 0.01) and supervisor feedback environment (Model 3: β = 0.22, *p* < 0.01). Hypotheses 1 and 3 receive full support. When adding supervisor identification to the model, supervisor identification also significantly predicts supervisor feedback environment (Model 4: β = 0.44, *p* < 0.05), but the effect of the feedback monitoring on supervisor feedback environment (Model 4: β = 0.17, *p* < 0.05) becomes lower.

**Table 3:** Hierarchical regressions for the impact of feedback monitoring/inquiry and supervisor identification on supervisor feedback environment.

	Model 1	Model 2	Model 3	Model 4	Model 5	Model 6
	Supervisor identification as dependent variable		Supervisor feedback environment as dependent variable			
Intercept	3.07^∗∗^	3.93^∗∗^	3.64^∗∗^	3.21^∗∗^	3.94^∗∗^	3.29^∗∗^
Feedback monitoring	0.35^∗∗^		0.22^∗∗^	0.17^∗^		
Feedback inquiry		0.24^∗∗^			0.16^∗^	0.12^∗^
Supervisor identification				0.14^∗^		0.17^∗^
Gender	-0.08	-0.07	0.01	-0.01	0.03	0.05
Age	0.09	0.06	0.01	0.01	0.02	0.04
Education	0.18^∗^	-0.23^∗^	0.17	-0.11	0.22	0.15
Tenure	0.10	0.14	-0.05	-0.1	0.02	-0.05
Δ*R*^2^	0.20	0.10	0.19	0.04	0.15	0.07
*F*	15.88^∗∗^	9.06^∗∗^	12.07^∗∗^	12.77^∗∗^	9.55^∗∗^	12.29^∗∗^


*n = 264; ^∗^p < 0.05, ^∗∗^p < 0.01*.

When the independent variable is feedback inquiry, as showed in **Table 3**, after controlling for the effect of participants’ demographics (gender, age, education, and tenure), feedback inquiry significantly predicts supervisor identification (Model 2: β = 0.24, *p* < 0.01) and supervisor feedback environment (Model 5: β = 0.16, *p* < 0.05). Hypotheses 2 and 4 receive full support. When adding supervisor identification to the model, supervisor identification also significantly predicts supervisor feedback environment (Model 6: β = 0.17, *p* < 0.05), but the effect of the feedback monitoring on supervisor feedback environment (Model 6: β = 0.12, *p* < 0.05) becomes lower.

To calculate the indirect effects, we adopt the SPSS micro PROCESS (Hayes, 2013). Bootstrap results in **Table 4** show that the indirect relationship between feedback monitoring and supervisor feedback environment through supervisor identification is significant (conditional indirect effect = 0.18, SE = 0.03, 95% CI = 0.02–0.16), and the indirect relationship between feedback inquiry and supervisor feedback environment through supervisor identification is significant (conditional indirect effect = 0.14, SE = 0.04, 95% CI = 0.02–0.07). Supervisor identification is partially mediated the relationship between feedback seeking and supervisor feedback environment. In sum, Hypothesis 5 and 6 is supported.

**Table 4:** Results of bootstrap for the indirect effect of feedback seeking on supervisor feedback environment via supervisor identification.

Dependent variable	Conditional indirect effect	*SE*	LL 95% CI	UL 95% CI
Feedback monitoring	0.18	0.03	0.02	0.16
Feedback inquiry	0.14	0.04	0.02	0.07


*n = 264. LL, lower limit; CI, confidence interval; UL, upper limit*.

## Discussion

Outcomes on antecedent variables influencing the feedback environment have been obtained, and organizations, tasks, supervisors, colleagues, and employees’ self-value systems are known to influence the feedback environment (Andrews and Kacmar, 2001); however, the above factors regard employees as passive accepting objects and ignore the initiative of employees in obtaining feedback. Limited research has introduced employee feedback-seeking to the formation of the feedback environment (De Stobbeleir et al., 2011) or discussed the forming mechanism of the feedback environment from the feedback-seeking angle. According to suggestions from Anseel et al. (2015), this research explores the influences of the follower-centered perspective on the feedback environment in terms of different feedback-seeking tactics. The results of this work present important significance to research on feedback theory and management practice. The purpose of this study is to examine the mediating effects of supervisor identification on the relationship between feedback-seeking and the supervisor-feedback environment. After controlling for the effects of demographics, the results show that supervisor identification partially mediates the relationship between feedback-seeking (including feedback monitoring and feedback inquiry) and the supervisor-feedback environment.

### Theoretical Contribution

In summary, this research presents the following theoretical contributions. First, it discusses the feedback environment formation mechanism from the follower-centered perspective by discussing the effect of feedback-seeking. This research finds that feedback inquiry and feedback monitoring positively influence the supervisor-feedback environment, and this result is consistent with the dynamic reversible model proposed by Anseel et al. (2015). According to the proposed model, low working performance can increase the feedback-seeking of employees and improve performance; the same logic is also applicable to other related variables, such as uncertainty. This reversible cause-and-effect relationship is verified by empirical research. According to Anseel et al. (2015), as followers of supervisors, the characteristic and the behaviors of employees can be taken as independent variables, and the characteristics and the behaviors of supervisors can be taken as dependent variables, with the concerns of the way for the characteristic, style, role orientation, identity, and behavior of employees to build the attitude of supervisors. Therefore, this research introduces the feedback-seeking into the forming factors of the feedback environment, and assumes that it is necessary for employees to seek for feedback to supervisors in ways of observation and inquiry to build preferable feedback environment for the creative performance with high uncertainty of correctness.

Second, our results add to the feedback literature by identifying supervisor identification as a mediating mechanism between different types of feedback-seeking and perceptions of a favorable supervisor-feedback environment. According to the results of this research, different tactics of feedback-seeking influence identification, and feedback monitoring exerts a significant influence on identity. This result is similar to the research findings of Sluss and Ashforth (2008), who observed significant differences between different feedback-seeking and identification. Considering that employees in China tend to save face when they communicate, indirect understanding and observation are the main forms influencing identification in this case. According to previous research findings, supervisor identification t2 plays a mediating role in the influence of feedback monitoring t1 and feedback inquiry t1 on the feedback environment. The confidence obtained from feedback-seeking will increase employees’ understanding of their supervisors, satisfy multiple self-motivations, and promote identity (Anseel and Lievens, 2007). As the return of identity, according to exchange theory, supervisors and colleagues can create an innovation-promoted feedback environment with higher quality.

### Practical Contribution

Firstly, the role of employee feedback-seeking should be studied, and supervisors should encourage feedback-seeking among employees and help them find a suitable feedback-seeking method to ensure that the feedback environment encourages employees to seek work-related help in an independent manner. Especially in working environments with fierce competition, employees can construct a better working environment if they can feel about more autonomous rights. Feedback-seeking provides more opportunities for employee management because it provide employees with more opportunities to understand the organizational target setting and performance expectations (Anseel and Lievens, 2007). By institutionalizing feedback behavior, managers are more likely to spend to provide feedback because employees know that the organization pays great attention to it.

Secondly, employees’ identification of a supervisor is very important because it involves in important working outcomes in and out of responsibilities. Because identification is regarded as an operation of interpersonal relations (Reich and Hershcovis, 2011), the focus of constructing fluid employee relationships is obtaining better performance targets and purpose method convention. Improving relations (manager–subordinate) will promote identity and the practice achievements, which is consistent with suggestions on adapting to a damaged performance management system (Pulakos and O Leary, 2011).

### Limitations and Future Research Suggestions

One of the disadvantages of this research is that although a longitudinal approach was utilized to determine the influence of feedback-seeking on the feedback environment, this research only pays attention to the outcomes of short-term work and does not take the complicated motivation and identity processes into consideration. Similar to feedback itself, the development of feedback is dynamic and changes over the long term. Thus, focusing on the relation between feedback-seeking and the feedback environment within several months is insufficient to complete elucidate the associations between these two concepts. Different feedback-seeking motivations can generate positive or negative outcomes. Analyzing the employee matching degree and feedback-seeking motivation by supervisors is a complicated identity process. Future research can take motivation factors into consideration to broaden the discussion on how motivation builds feedback-seeking behaviors and influences the ways the feedback information takes effect. A discussion on the awareness of supervisors toward feedback-seeking motivations will also influence on supervisors’ opinions on the feedback environment (Dahling et al., 2015).

Second, this research only controls genders, ages, length of service, and degree of education but does not distinguish or control personalities, job levels, job transfer or assumption of duty, enterprise scale, and post-nature. According to research findings, goal orientation and personality are factors that impact the feedback process. Therefore, future research should consider other factors influencing the causal relation between these two concepts while discussing the focal problem. Variables such as goal orientation, personality characteristics, employment, service experience, and work nature should also be taken into consideration to increase the universality of the research conclusions.

Third, this research does not determine the full mediation effect of supervisor identity in the relation, which means other factors, could influence the feedback mechanism. Thus, future research could explore other mediating mechanisms connecting these two factors. For example, the hypothesis that the effectiveness of individual belief feedback is valuable to achieve the working target may be an important factor influencing the feedback mechanism. Several other factors, such as whether the change in behavior after identification is in accordance with the standards of the supervisors, may influence the relation between identification and the feedback environment. Such factors may be discussed in future research.

## Author Contributions

ZG provides substantial contributions to the research conception and design. YQ and NZ analyzes and interprets the data. ZG and ML write the paper.

## Conflict of Interest Statement

The authors declare that the research was conducted in the absence of any commercial or financial relationships that could be construed as a potential conflict of interest.
